# Editors’ Best of 2024

**DOI:** 10.1016/j.jaacop.2024.12.003

**Published:** 2024-12-30

**Authors:** Robert R. Althoff, Kara S. Bagot, Joseph Blader, Daniel P. Dickstein, Robert L. Findling, Manpreet K. Singh

## Abstract

In our second year as *JAACAP Open*, which is now available on PubMed Central, we are proud to support the dissemination of among the highest quality research being conducted in our field. Choosing the “best” among stars is always a tall order and most certainly misses the many ways that articles make an impact: is the “best” the most interesting, the most surprising, the most educational, the most important, the most provocative, or the most enjoyable? How do we decide? This time around, our team made some picks based on those that were methodologically sophisticated, attuned to the complexity of childhood-onset psychopathology, and clinically salient. It is our pleasure to give a special “hats off” to the 2024 articles that we think deserve your attention or at least a second read!

In our second year as *JAACAP Open*, which is now available on PubMed Central, we are proud to support the dissemination of among the highest quality research being conducted in our field. Choosing the “best” among stars is always a tall order and most certainly misses the many ways that articles make an impact: is the “best” the most interesting, the most surprising, the most educational, the most important, the most provocative, or the most enjoyable? How do we decide? This time around, our team made some picks based on those that were methodologically sophisticated, attuned to the complexity of childhood-onset psychopathology, and clinically salient. It is our pleasure to give a special “hats off” to the 2024 articles that we think deserve your attention or at least a second read!

### Executive Functioning, Internalizing and Externalizing Symptoms: Understanding Developmental Dynamics Through Panel Network Approaches, Freichel *et al.*

How can we capture the complex interplay between psychiatric disorders in childhood and adolescence? How do we describe and explain the common co-occurrence of mental disorders that we observe in youth? How can we make sense of the heterotypic relations between disorders that may begin one way in youth but develop very differently throughout adolescence and into adulthood? Like the answer to many a dad joke, the answer is “very carefully.”

Freichel and colleagues^1^ approach the complex interplay between executive function, internalizing, and externalizing disorders over time by taking a very careful approach in a unique longitudinal sample of Dutch youth. In this analysis of the Tracking Adolescent’s Individual Lives (TRAILS) survey, they used 3 assessments of more than 1,600 youth from ages 10 to 16 years, including neuropsychological and cognitive assessment at baseline, and applied 2 unique types of analyses to account for co-occurrence of multiple symptom types and the longitudinal course. In the first models, they used cross-lagged panel network (CLPN) models to examine age effects within and across waves while accounting for within- and between-person effects. They also used panel graphic vector autoregressive (GVAR) models to look at the overall network structure of the symptoms, but taking into account within- and between-person effects. Taking this careful and rigorous approach, they demonstrated the central role for depressive symptoms interconnecting many of the other internalizing symptoms with externalizing symptoms with multiple reinforcing feedback loops between internalizing and externalizing disorders. They further demonstrate sustained attention as an underlying transdiagnostic risk factor. Reviewing these data, one begins to understand the dilemma of the late adolescent or young adult patient coming into the office with concerns that “No one has figured out my diagnosis. They keep telling me something different.” With continual feedback among disorders, there is likely to be frequent and complex back-and-forth among symptom clusters. Thankfully, there is careful, innovative science like this work from Freichel and colleagues to demonstrate why this may be the case. And this care and innovation make this paper one of our “Best of 2024.”

**Robert R. Althoff, MD, PhD**

### Review: Preventing Psychopathology in the Digital Age: Leveraging Technology to Target Coping and Emotion Regulation in Adolescents, Henry and Compas

The global burden of psychiatric illness among youth 10 to 24 years of age is 45%,^2^ with onset of mental illness by ages 14, 18, and 25 years estimated to be 35%, 48%, and 63% respectively.^3^ In the United States, nearly 50% of adolescents 13 to 18 years of age will receive a lifetime psychiatric diagnosis^4^; however, fewer than 20% of adolescents in the United States are engaged in mental health care.^5^ Although there has been some evidence of increasing engagement in mental health care over the past 20 years among adolescents in the United States, particularly among those with internalizing disorders, there have been temporal decreases in care among minoritized groups, particularly non-Hispanic Black adolescents, those of low socioeconomic status, and those with public insurance (ie Medicaid, Children’s Health Insurance Program [CHIP]).^5^ Technology-based interventions may address barriers to early intervention, including cost, stigma, and access, and may increase overall reach and scalability of traditional primary and secondary prevention interventions. Furthermore, because of the ubiquity of ownership of mobile phones among African American (94%) and Hispanic (98%) youth,^6^^,^^7^ app-based interventions have the potential to decrease health disparities for minority youth who, in addition to having lower access to care, suffer from greater adverse consequences due to the burden of mental illness.^8^ Despite this potential, there remains a huge gap between technological advances used by youth, the capacity for technology to have a positive impact on health care intervention delivery and accessibility, and evidence-based technological resources for health behaviors.

In the article “Review: Preventing Psychopathology in the Digital Age: Leveraging Technology to Target Coping and Emotion Regulation in Adolescents,” Henry and Compas^9^ review the literature related to technology-based/enhanced interventions for prevention of psychiatric illness in youth with a focus on coping and emotion regulation. The authors find that coping strategies targeting cognition and attention, physiology, and behavior, and those strategies associated with more psychopathology (eg, thought suppression, expressive suppression, avoidance), may be most effective in improving emotion regulation in stress response. Barriers to traditional treatment including access, stigma, fidelity, and maintenance of gains, which may also be related to retention in treatment and other such barriers, are also discussed and can be generalized across mental health treatment.

This review highlights the limitations of the current state of the science regarding technology-based approaches to intervention in psychiatric illness, including lack of industry–healthcare partnerships, limited guidance on establishment of such partnerships, the dearth of studies examining fidelity of in-person to technology translated interventions, the cost of developing such interventions, and the lack of an evidence base on dissemination and implementation of technology-based mental health intervention. The authors also highlight the nascent evidence base for primary prevention interventions focused on coping strategies in youth, lack of implementation of evidence-based interventions in health care settings, especially those settings for youth or community-based settings, and under-identification of mechanism of action of efficacious and effective interventions limiting the ability to translate these interventions to alternative settings, populations or modes of delivery. Although studies suggest that we may increase engagement in, and effectiveness of, primary and secondary prevention interventions by developing selective, highly specific interventions that include content that addresses salient and developmentally appropriate risk factors for youth that are presented on interactive platforms, few interventions have done so, despite evidence that youth are digital natives. Although mobile and digital technologies remain highly promising and palatable intervention platforms for youth, much research remains to be done to effectively use this medium to reduce the mental health burden of youth.

**Kara S. Bagot, MD**

### Sex-Specific Depressive Symptom Trajectories Among Adolescents in Los Angeles County, 2013 to 2017, Gimbrone *et al.*

Well-done research can be intriguing for any number of reasons, but I perk up for work that integrates into one rubric what seems at first like distinct areas. Gimbrone and colleagues’^10^ contribution weaves together 3 incompletely^11^ understood, albeit clinically vital, aspects of depression: (1) people with ostensibly the same diagnosis show variability in course and outcomes^12^; (2) there is a sex disparity in the rates of depression after childhood, with female individuals showing greater prevalence worldwide^13^^,^^14^; (3) since the turn of the 21st century, depression has been on the rise, especially among young female individuals.^15^^,^^16^

Gimbrone *et al.*’s article’s innovation is the use of empirically derived trajectories in adolescents’ reports of depression symptoms to identify different patterns in longitudinal courses between female and male individuals. The results should catalyze further research on whether these sex-specific trajectories may themselves change over time, perhaps influenced by the same forces that produce shifts in the prevalence and other features of mood disorders recently observed in many countries.

In the fall of 2013, the investigators recruited a cohort of 3,393 ninth graders from 40 high schools in Los Angeles County who then participated in 8 biannual assessments that included the self-reported Center for Epidemiological Studies—Depression scale (CES-D). Retention was a remarkable 99.3%. These longitudinal data enabled the use of semiparametric growth mixture modeling to derive latent classes of trajectories. This contrasts with the typical presentation of longitudinal data, which shows the mean at each time point, ignoring the huge variability in individual trajectories that the single average curve belies. Instead, mixture models for growth curves derive a set of prototypic curves that represent subgroups of participants whose trajectories better resemble these specific patterns than the overall group trend. Gimbrone *et al.* looked at whether fitting trajectories for male and female participants separately better fit the observed longitudinal patterns than a group of trajectories that ignored sex.

Trajectory classes (depicted in Figure 1) showed both similarities and differences between male and female participants, with stratification by sex providing better overall fit. Both sexes had large groups that reported low CES-D scores at all time points (“low stable,” 35% of female and 49% of male participants) and a group that showed decreases from their initial, elevated scores, but that nevertheless remained high during the follow-up period (“decreasing,” 16% of female and 12% of male participants). But whereas female participants contained a trajectory group that was consistently in the low clinical range (“mild stable,” 43%), male participants had a group that crept up into the clinical range by their junior year (“mild increasing,” 35%). Table 3 shows that the slope for male participants in this group was nearly double that of female participants. Male and female participants also each had a group with consistently quite high ratings. However, among female participants, this group’s scores peaked in 10th grade and then declined a bit (“high arching,” 6%), whereas among male participants there was a steady rise that by senior year essentially matched that of female participants (“high increasing,” 4%).

Comparing their findings to similar studies conducted before 2010, the authors report that their overall prevalence of elevated depression ratings are higher, and that membership in the trajectory classes showing increases is also larger. However, coming from a single US county, generalizations to other populations and to other time periods can be only tentative.

These results, elegantly presented, merge findings on overall sex differences in depression prevalence with distinct temporal patterns in severity to offer a more nuanced picture of adolescent depression. The results confirm female individuals’ higher susceptibility to depression symptoms, and variability in trajectories may identify subgroups to which its some proposed explanations apply more than others.^13^^,^^14^ Likewise, evidence for rising symptom incidence among male individuals over the high school years, if robust and replicated, is both concerning and underappreciated. They are also relevant to the discussion since the 1990s about social changes that purportedly have had negative impacts on, among other things, young male individuals’ identity, economic success, and educational attainment—issues that may become more salient for male adolescents as the end of high school approaches.

Time and further research will tell whether sex effects in the incidence and course of mood and other problems are influenced by such broader sociocultural trends. When they do, I will, with appreciation, keep in mind the role that Gimbrone *et al.* played in fostering a more nuanced approach to an area that is central to the promotion of adolescent well-being.

**Joseph C. Blader, PhD**

### Longitudinal Pathways From Maltreatment to Substance Use Through Delay Discounting During Adolescence and Into Young Adulthood, Peviani *et al.*

Childhood maltreatment is a preventable exposure that is all too common and linked to substantial morbidity and mortality. According to the Centers for Disease Control and Prevention’s (CDC’s) Youth Risk Behavior Surveillance Survey (YRBS) 2023 of high school students in the United States, approximately 3 of 4 high school students in the this country report experiencing at least 1 adverse childhood experience (ACE), with 18.% experiencing 4 or more ACEs, the most common being emotional abuse (61.5%), physical abuse (31.8%), and household poor mental health (28.4%).^16^ Moreover, a study of 49,853 adults in the Collaborative Perinatal Project showed that participants who experienced family instability had greater risk of dying by suicide and substance use.^17^

Despite knowing all of the above, it is important that understanding the trajectory from maltreatment exposure to substance use behaviors remains unclear. Enter Peviani and colleagues’ article “Longitudinal Pathways from Maltreatment to Substance Use Though Delay Discounting During Adolescence and Into Young Adulthood.”^18^ This study examined data from 167 adolescents (53% male, mean baseline age 14 years) who provided longitudinal data about substance use, including cigarette, alcohol, and cannabis frequency, at 5 time points between ages 14 and 18 years.

During those same timepoints, they also provided information about delay discounting—defined as the preference for small immediate rewards over greater rewards received later in time. In this study, it was assessed using a series of hypothetical decisions about whether they would rather pick a smaller reward sooner compared to a larger award later. This is a variant of the classic Popeye cartoon character Wimpy’s motto “I’ll gladly pay you Tuesday, for a hamburger today.”

This study added yet a third element, asking about participants’ exposure to maltreatment during ages 13 to 17 years when the participants were ages 18 to 19.

Key findings from this study were that adolescents exposed to neglect were especially vulnerable to cannabis use over time, mediated through increased delay discounting. Moreover, neglect experiences predicted greater cigarette use over time.

From this, the authors conclude that delay discounting offers an important window into prevention and intervention opportunities for how to reduce the risk of substance use among adolescents who have experienced neglect. The authors discuss this in relation to the Dimensional Model of Adversity and Psychopathology, in particular how this finding is about neglect, or maltreatment by omission, rather than about abuse, or maltreatment by commission. Taken as a whole, this article suggests that we should not delay our efforts geared toward translating these findings into novel treatments and risk reduction strategies.

**Daniel P. Dickstein, MD**

### Relative Age Effects on Attention-Deficit/Hyperactivity Disorder Symptoms and Educational Achievement: A Longitudinal UK Cohort Study, Deng *et al.*

There have been several papers that have reported that school-aged children who are younger than most of their classmates (“young-in-class” students) do less well than their “old-in-class” peers, both educationally as well as in other domains during childhood. This has been given the name “relative age effect.” For example, there are reports that suggest that a relative age effect may place children who are younger than their classmates at risk for both receiving the diagnosis of attention-deficit/hyperactivity disorder (ADHD) as well as risk for academic underachievement.

In *JAACAP Open*, Deng and colleagues’^19^ paper tested several hypotheses using data from a longitudinal cohort from England and Wales. The Twins’ Early Development Study (TEDS) evaluated twins born between 1994 and 1996 and evaluated this cohort until they became adults. Children in this cohort were followed from age 7 years to age 21 years. This longitudinal methodology sets itself apart from several prior reports that reported on cross-sectional associations and allowed for several outcome variables to be evaluated over time. It should be mentioned that this was a relatively large study with almost 4,000 young-in-class pupils and more than 4,500 children who were not young-in-class.

The authors found, based on parent report, that there was a relative age effect on the amount of ADHD symptoms reported at the initial measurement timepoint (8 years of age). The investigators also reported that younger children appeared to have more ADHD symptoms during middle childhood, but that they did not necessarily have more ADHD symptoms later during adolescence. Of note, for young-in-class students, reduction of ADHD symptom scores might decrease to a greater degree over time, when compared to those of children who were initially old-in-class. In addition, the authors noted that at age 15 to 16 years, lower standardized test scores were found in English but not mathematics for the young-in-class subcohort.

Finally, it was evaluated whether or not ADHD polygenic scores (PGS) would be less strongly associated with ADHD symptoms for those who were young-in-class vs their older classmates. The author found that ADHD PGS was not less strongly associated with younger versus older classmates.

This paper, voted a “Best Of in 2024,” provides evidence to suggest that children who are young-in-class may be at increased risk for both increased ADHD symptoms as well as educational outcomes that are worse than those of their peers. As this is a group of children who might have a greater likelihood for poorer outcomes than other children, means by which to support these young-in-class children, once identified, might lead to improved outcomes for this potentially vulnerable population.

**Robert L. Findling, MD, MBA**

### Prospective Follow-up of Adolescents With and at Risk for Depression: Protocol and Methods of the Identifying Depression Early in Adolescence Risk Stratified Cohort (IDEA-RiSCo) Longitudinal Assessments, Piccin *et al.*

Not too long ago, society did not believe that children could be sad or could experience sustained periods of sadness that could constitute a depressive episode. With increased recognition of depression phenotypes and how they present across various stages of development and in various cultures, there is much to understand and document about depression and the risk factors leading up to when it first manifests. In the year’s Best of *JAACAP Open* 2024, Piccin and colleagues^20^ describe the protocol and methods of a comprehensive set of prospective assessments of youth in Brazil at risk for depression. The protocol implemented the most current clinical and digital phenotyping tools available to assess depression and risk for depression among 7,720 adolescents screened in Brazil. Of those screened, 75 boys and 75 girls were recruited and followed for 3 years and assessed using clinical, parent, and self-reported measures, and through active and passive data sensing via smartphones. In addition to these and other ecological momentary assessments, Global Positioning System (GPS) data as well as neuroimaging and bioassays were collected, with excellent retention. Particularly novel was the use of a WhatsApp bot, as the application is widely used in low- to middle-income countries, which facilitated the collection of information from diverse sources in a longitudinal design, demonstrating that adolescents around the world can feasibly be recruited into research over an extensive period. This protocol highlights several important priorities for our field. First, the approach allowed for deep phenotyping to advance understanding of depression in young populations, particularly in areas where mental health research is scarce. Second, by collecting these data in previously uncharacterized youth, we may in the future be able to determine shared and distinct phenotypes of depression between youth and adults, and between youth from Brazil versus youth from other parts of the world. Third, and perhaps most noteworthy, identifying early risk factors for depression will facilitate the development of early interventions that are culturally and contextually sensitive. Thus, this innovative protocol provides hope for a future in which the global burden of depression among youth is less than it is today.

**Manpreet K. Singh, MD, MS**
